# Syphilitic Cachexia

**Published:** 1908-05-30

**Authors:** Dyce Duckworth

**Affiliations:** Consulting Physician to St. Bartholomew's Hospital; Senior Physician to the Seamen's Hospital, Greenwich.


### Hospital Clinics.

### SYPHILITIC CACHEXIA.

By SIR DYCE DUCKWORTH, M.D., LL.D., F.R.C.P., Consulting Physician to St. Bartholomew's Hospital; Senior Physician to the Seamen's Hospital, Greenwich.

(A Clinical Lecture delivered at the London School of Clinical Medicine.)

In this hospital we are sadly familiar with many of the conditions induced by venereal infection, and my clinic here is seldom free of patients illustrating those phases of the disease which demand medical rather than surgical treatment. The reason for this is not far to seek. Seafaring men are more than others liable to this foul infection, contracted in various parts of the world, often under the influence of alcohol, and in ignorance of, or in indifference to, the serious risks run by indulgence in riotous passions.

Infection having been incurred, these men proceed again to sea, untreated, or often most imperfectly treated, for any symptoms of the disease that may present themselves. Even in the best class of vessels where competent advice is provided, the surgeon has seldom an opportunity of carrying out the essential and prolonged course of treatment which such cases imperatively demand, since these patients disappear and re-embark on other voyages, having made but temporary and incomplete recovery.

When, during last session, I was discussing with some of you the treatment of cirrhosis of the liver, I laid stress on the differences observable in the cases of that disease as occurring in the upper and lower classes of society, the latter for several reasons presenting the worst forms of it. We find in the case of syphilis that the lower orders, and particularly seamen, suffer more severely, and exhibit the gravest sequels, in greater proportion than those who are well placed in society, and are therefore enabled to secure prompt and adequate treatment. Of course, I am now alluding to our merchant seamen, for we know that our sea and land forces receive due and skilled attention after infection, although our sentimental Legislature is serenely indifferent to the adoption of proper measures for the prevention of such infection in all parts of our empire.

We, in our profession as "priests of the body," claim to have as high and Christian conceptions in regard to the working of any Contagious Diseases Acts as the most vehement agitators against them profess to hold, but we will not keep silence while no endeavours are made to mitigate or remove the spread of a noxious and devitalising malady throughout the Empire. We leave to the "priests of the soul" the duty of uplifting the morals of our communities and reinforcing our efforts to promote purity and self-control.

I have nothing to say respecting what are called the primary and secondary stages of syphilis. For some occult reason the surgeon is supposed to have to do with these phases. I will, however, remind you of the occasional occurrence in these stages—that is, within three months, or even more, of primary infection—of a peculiar remittent or intermittent fever which may, if not recognised or thought of, suggest the onset of enteric or other infection. I have little doubt that such cases at sea and elsewhere are sometimes ascribed to tropical malaria, and prove a source of difficulty in diagnosis, being rebellious to any but antisyphilitic treatment. My remarks to-day relate to the tertiary or latest phases of venereal disease, to which the term syphilitic cachexia is applied. I prefer this term, for it directs attention to the actual state of the whole organism, the blighted and impoverished nutrition, the anæmia, and the constant liability to gross structural changes in the bones, viscera, nervous system, and arterial tunics. As you already know, it is not easy to say when the secondary term of venereal manifestation passes into the third. We usually note the change in the character of the skin eruptions at the later period, passing from macular, papular, or scaly forms on the surface into deeper and ulcerating lesions, which leave permanent scars with pigmentation round them. The presence of gummata is a plain indication of the tertiary stage, and you are familiar with their occurrence in the skin, periosteum, muscles, and internal organs.

In the skin these tend to break down into ulcers of an obstinate character. In the heart and liver they are apt to undergo fibroid change, which leads to puckering and massive deformities. On mucous surfaces, again, the tendency is to ulceration, leaving cicatrices after healing which lead to serious strictures in the larynx or the rectum, or to destruction of the palate and nasal bones.

Amongst the later developments we sometimes find amyloid or waxy degeneration widely spread in the body, affecting especially the liver, spleen, intestinal mucosa, and the kidneys. With respect to this particular change, I have to record my experience to the effect that it is now clearly a much rarer sequel of syphilis than it was in my student days, and I believe this fact to be due to the better treatment of the earlier stages of the disease, and, in part, to the infection itself being generally of a less virulent quality than it was wont to be. Amyloid degeneration, as a result of chronic suppuration of any variety, is also now far less frequently met with since modern surgery has been enabled to arrest the process of rebellious depuration which was formerly so common and fatal.

Patients who suffer from syphilitic cachexia are, as a rule, recognised by their general aspect. They are pallid and sickly looking, ill nourished, lean, and feeble. The several lesions already mentioned may be found in the affected parts. Cases occur, however, in which these specific changes are not readily discovered, and many mysterious symptoms may be presented which are puzzling to the observer. So much so is this the case that if in any male patient in the third or fourth decade of life strange and unaccountable symptoms occur, it is always wise to bethink oneself of the possibility of syphilis as the fundamental source of them, and to act on such an assumption, whether a history of past infection is forthcoming or not. Not seldom, in such instances, does appropriate medication tend to clear up a difficult diagnosis. We are sometimes shocked by sudden and unexpected deaths in persons in middle life who have not complained of any noteworthy symptoms. A necropsy reveals the cause, and a gumma is found in the myocardium, which led to the fatal syncope on some trifling exertion, altogether unsuspected and untreated. In these cases, as I have remarked, there may be little complaint of cardiac disturbance, and such symptoms as may occur, if inquired into, are only too likely to be set down to some functional disturbance. Here, again, we should always bear in mind, in the case of apparently robust men of middle age, the terrible possibility of this particular and insidious lesion, and apply appropriate treatment.

Gummata occur in the brain and spinal cord, chiefly in the membranes, inducing meningitis, but they may be found as gross lesions in the brain and cord, producing all the symptoms of tumours, sometimes associated with encephalitis or myelitis. The arterial system is very apt to suffer. Syphilis is well recognised as one of the main destroyers of arteries. Arteritis, with obliteration of the lumen of the vessel, is common (endarteritis obliterans) or gummatous periarteritis, especially frequent in the cerebral arteries.

It appears likely that the majority of aneurysms of the aorta, certainly those in women, are the result of former infection by syphilis, aggravated too often in seamen and soldiers by strain and the effects of alcoholic excess. Gummata may arise in the testes and give rise to an interstitial orchitis. The kidney may also be the site of gummata, and very rarely they may occur in the lung. Syphilitic phthisis has been described, but I have never met with such a case. The changes in the bones are readily recognised by painful nodes and enlargements, sometimes with gross deformities, commonly in the tibiæ and femora, but also met with on the skull, clavicles, scapulæ and sternum.

Changes may occur in the joints as a result of tertiary syphilis. Gummatous infiltration with effusion may be found, apart from any bony change. Synovial thickening with marked fimbriation and pitting of the articular cartilage extending to the bone is recognised as a variety of syphilitic arthritis. Another form appears to arise in the bone and spread to the joint, associated with effusion. I have little doubt that some of the obstinate forms of arthritis we meet with in this clinic are of syphilitic, and not always of gonococcal, origin. In the alimentary canal we find the stomach very seldom involved. Ulceration in the small intestine is also rarely met with. The rectum is a common site, in its lowest portion, for ulceration, and sclerosing changes leading to stricture are a frequent sequel.

In all cases we have to regard the personal factor. The effects of syphilitic infection vary according to the special constitution and proclivities of the patient. The worst subjects for the disease are those of a scrofulous habit of body, and many of the gravest cases are thus complicated by this inherent vulnerability. Alcoholic subjects are also apt to suffer more than others. Naturally, persons of robust constitution offer the highest resistance to its inroad and development, and recover best from its ravages if adequately treated. Again, we have to note the strange latency pertaining to venereal infection in its later stages, indicated at times by long apparent arrest and quiescence.

A failure in general health, or the onset of other ailments, is apt in such cases to lead to a revival of the various lesions due to the original infection. The same condition, you will remember, is noted in cases of malarial impregnation. The peccant matter lies dormant, as it were, ready to be aroused into activity by any causes inducing trophic depression. We may feel sure that such occurrences are significant of an incomplete treatment at the outset of the malady, and that sound health has never really been restored. A long and wide experience now enables us to speak of a radical cure in the majority of cases of syphilis, provided that a thoroughly adequate and prolonged course of treatment has been carried out, and thus we ought rarely to meet with examples of the cachexia we are considering to-day. In fact, we now see fewer of these cases than we were wont to do, owing to more intelligent treatment and a generally higher standard of hygiene and cleanliness than was formerly practised.

I must refer to those disorders termed para-syphilitic, which may be regarded as sequels of the original infection, since they are commonly met with in subjects who were once the victims of syphilis. I allude to locomotor ataxia, a variety of paralytic dementia, some forms of epilepsy, and a generalised arterio-sclerosis. None of these are to be regarded as direct syphilitic manifestations. They certainly do not respond to the treatment for syphilis, and yet we have to recognise that the original infection has distinctly prepared the way for these maladies, inducing blighted areas of nervous matter, and undermining their delicate mechanism and normal metabolism. They illustrate only too well some of the terrible consequences and outcome of this devastating and insidious disease, and enforce the lessons not only of social preventive measures, but the serious importance of dealing adequately with it in its earliest stages.

To these it is not now for me to allude in any detail, and I will add no more than the expression of my firm belief in the specific virtues of mercury as the sole agent for eradicating the poison of *lues venerea* known to us. We know well that to secure the full benefit of this treatment it must be carried out carefully for at least two years. The failure to conduct it properly over so long a period accounts for the many relapses and the eventual onset of the graver conditions we are discussing to-day. Hence, we find the larger proportion of these in our lower orders, and especially in seafaring men who are unable to secure adequate and sustained treatment, or who carelessly neglect the advice given to them. The case is very different in subjects of the better-placed classes, who are submissive to this prolonged medication, and aware of the dangers of neglect to carry it out, for in these persons we rarely meet with serious relapses or recrudescence of any manifestation of the disease in after life, and they are able to undertake all the duties of life, and capable of founding healthy families. No syphilitic cachexia is met with in such cases, and no parasyphilitic affections are apt to supervene. Doubtless we may meet with instances of the latter in the higher classes of society, but they are commonly examples illustrating a local infection which has been overlooked at the outset, unsought, or long forgotten, but nevertheless specific and potential; with all the liabilities to further developments, yet untreated from the beginning. It is true that in spite of thorough and persistent treatment, some few cases occur in which the dregs of the disease, as it were, still manifest themselves in later life, and it has been noted that where the tokens of the secondary period have been but slightly developed, there is some tendency for the later visceral lesions to supervene more severely. The reason for this is probably to be found in the fact of the milder secondary symptoms having led to the opinion on the part of the patient and his adviser that the original infection was either very mild or had already been overcome.

The specific value of mercury is, as I have said, well recognised. Forty years ago there was a strong prejudice against this form of medication, and this arose from the fact that it had been formerly much abused, and employed in large doses, which induced such degradation of the tissues and devitalisation as to add a mercurial cachexia to the evils already present. The reaction was great, and the Edinburgh school in particular took a leading part in it. The mischief that ensued was not long in manifesting itself, and mercury again came into vogue, this time properly and moderately employed. Iodide of potassium, and iron, which had replaced mercury for the time being, were not found to be adequate to destroy the insidious taint, nor were any other methods which supplanted mercury in the earlier stages of syphilis.

In the treatment of the later stages, when gummata and visceral disease are present, we know of no better remedy than the iodide salts. Their action is prompt and decisive. So much so is this the case that these agents have come to be regarded as a touchstone, so to speak, for any lingering venereal taint. This is not to be taken as infallible, however, for iodide of potassium has resolvent powers over other than syphilitic growths. In the cachexia of syphilis, then, we cannot dispense with the iodides. They are to be given in doses of from 3 to 20 grains three times a day. Sometimes even larger doses may be required for a time.

We sometimes find the iodides productive of depressing effects. This is more liable to occur with the potassium salt. In such cases we may replace it by the sodium or ammonium salt, or give all three together. A favourite prescription of mine consists of such a mixture with the compound tincture of cinchona (Huxham's tincture) and a full dose of the concentrated compound decoction of sarsaparilla. I must add a few words on the value of the latter drug, for it has largely gone out of use, and I have a high opinion of it. My former master, the late Professor Syme, of Edinburgh, was wont to say that "it was of no more use than a decoction of so much hay." I doubt greatly if he had ever employed it in appropriate doses before he ceased to use it.

The ordinary compound decoction in doses of 1 ounce as prescribed, if prescribed at all, has little value beyond its pleasant flavour. This remedy was in favour long ago in Germany in the two forms of Zittman's decoction—*fortius and mitius*—(Pharm. Borussica 1847). It resembled the compound decoction of our present Pharmacopœia in several respects, but it contained a little mercuric perchloride or, perhaps, cinnabar, and was given as a sort of diet-drink in doses of as much as a pint in the day. Some may be disposed to attribute the virtues of this medication to the mercury alone, but those who have employed sarsaparilla in adequate doses, apart from mercury, are well-satisfied of its powers in removing cachectic conditions due to syphilis and other morbid states.

Not less than half-ounce doses of the concentrated compound decoction should be given three times a day, and it can be well administered by itself from time to time in such cases as we are considering to-day, or occasionally with the Liq. Hydrarg. Perchloridi, or with the iodides, or with both of these, always remembering in chronic constitutional ailments the *dictum* of Trousseau, that great clinician, that "chronic diseases demand chronic therapeutics." We so frequently half-treat our patients that it is well to bear this teaching in mind.

The results of the treatment I have commended to you are commonly satisfactory, and sometimes very remarkable. We note the disappearance of gummata in the viscera, bones and skin, reduction in enlarged nodular livers, improvement in the richness of the blood, and a return of body-weight, vigour and well-being. The progress extends over months, not weeks, but the power we possess in following up this line of treatment rescues many of these sadly stricken patients from a life of misery and unceasing decay, and it is worth any efforts to secure such benefits.

The lecture was illustrated with specimens from the museum, and a patient, a marine engineer, was exhibited who had been operated upon for ascites a year previously in the provinces, and was found to have a large tumour on the under surface of the liver. After his admission in April the ascites continued, and the abdomen was twice tapped. A few weeks later the left lobe of the liver became nodular with new growths. There was albuminuria and œdema of the legs. The chance of recovery appeared very small. The treatment consisted of perchloride of mercury, iodide of potassium, cinchona, and full doses of sarsaparilla. The general health began to improve, the ascites, œdema, and albuminuria passed off. The gummatous nodules in the liver, the ascites, œdema, and albuminuria disappeared, and the patient regained weight and vigour. There was no clear history of primary syphilis or alcoholism. This patient hopes soon to return to duty at sea, after seven months' stay in the hospital.

